# Effect of KNO_3_ on Lipid Synthesis and CaCO_3_ Accumulation in *Pleurochrysis dentata* Coccoliths with a Special Focus on Morphological Characters of Coccolithophores

**DOI:** 10.7150/ijbs.35664

**Published:** 2019-11-01

**Authors:** Xuantong Chen, Ayyappa Kumar Sista Kameshwar, Chonlong Chio, Fan Lu, Wensheng Qin

**Affiliations:** 1 Department of Biology, Lakehead University, 955 Oliver Road, Thunder Bay, Ontario, P7B 5E1, Canada; 2 Faculty of Natural Resources Management, Lakehead University, 955 Oliver Road, Thunder Bay, Ontario, P7B 5E1, Canada; 3 School of Civil Engineering, Architecture and Environment, Hubei University of Technology, Wuhan, China, 430068

**Keywords:** *Pleurochrysis dentata*, Coccolithophore, Calcium, Lipid, Carbohydrate, Calcium carbonate (CaCO3), Potassium nitrate (KNO3).

## Abstract

*Pleurochrysis* genus algae are widely distributed in ocean waters. *Pleurochrysis sp.* algae are popularly known for its coccolithophores. Calcium carbonate (CaCO_3_) shells are major components of the coccolithophore, and they are key absorbers of carbondioxide. In this study, we have reported the effects of potassium nitrate (KNO_3_) concentration on calcium accumulation and total lipid, carbohydrate and protein contents of *Pleurochrysis dentata.* Results obtained from complexometric titration and scanning electron microscopy analysis showed higher rates of CaCO_3_ accumulation on *Pleurochrysis dentata* cell surface*.* We have also observed that overall cell size of *Pleurochrysis dentata* reached maximum when it was cultured at 0.75 mmol L^-1^ of KNO_3_. During 10 days of *Pleurochrysis dentata* culture total lipids and carbohydrate contents decreased, with slightly increased protein content. Results obtained from Fourier-Transform Infrared Spectroscopy (FTIR) also reported an increase in protein and decrease in lipids and carbohydrate contents, respectively. Similarly, *Pleurochrysis dentata* cultured at 1 mmol L^-1^ concentration of KNO_3_ exhibited the lowest carbohydrate (21.08%) and highest protein (32.87%) contents. Interestingly, *Pleurochrysis dentata* cultured without KNO_3_ exhibited 33.61% of total lipid content which reduced to a total lipid content of 13.67% when cultured at 1 mmol L^-1^ concentration of KNO_3_. Thus, culture medium containing higher than 1 mmol L^-1^ of KNO_3_ could inhibit the cell size of *Pleurochrysis dentata* and CaCO_3_ accumulation in shells but it could promote its cell growth. For the first time we have reported a relatively complete coccolith structure devoid of its protoplast. In this study, we have also described about the special planar structure of *Pleurochrysis dentata* CaCO_3_ shells present on its inner tube of the R unit and parallel to the outer tube of the V unit which we named it as “doornail structure”. We believe that this doornail structure provides structural stability and support to the developing coccoliths in *Pleurochrysis dentata*. Also, we have discussed about the “double-disc” structure of coccoliths which are closely arranged and interlocked with each other. The double-disc structure ensures fixation of each coccolith and objecting its free horizontal movement and helps in attaining a complementary coccolith structure.

## Introduction

Increasing population and industrial revolution are responsible for global fossil fuel consumption. Fossil fuel consumption leads to release of greenhouse gases into the atmosphere, which further leads to climatic conditions such as droughts; heat waves altered rainfall patterns raising sea levels and increasing global temperatures. Carbon dioxide (CO_2_) is the most important greenhouse gas; increasing CO_2_ concentration leads to global warming, ocean acidification and hypercapnia (caused due to increased CO_2_ in ocean waters). Ocean water is natural sinks for CO_2_; studies have reported that ocean water absorbs about 40% of global carbon dioxide through anthropogenic activities 1. However, the dissolved CO_2_ transforms to carbonic acid which further dissociates into bicarbonate and hydrogen ions resulting in ocean acidification (reducing ocean water pH) a major threat to the marine ecosystem 2, 3. Marine animals have developed different strategies to protect the cells from hypercapnia and lowered pH 4, 5. Different species and groups of marine animals such as molluscs, crustaceans, echinoderms have incorporated calcium carbonate into their skeletons. Increased carbon dioxide consumption and ocean acidification significantly affect the calcification process and calcium carbonate impregnation in shells or plates of marine animals and planktonic algae (Ca^2+^ + HCO_3_^-^ → H^+^ + CaCO_3_) 6.

Coccolithophorids are abundant phytoplanktons, majorly observed tropical and sub-tropical offshore waters, and are responsible for production of a large part of modern oceanic carbonate 6. Coccolithophores are well-studied planktonic algae for their effect on global carbon cycle. Coccolithophores classified into division haptophyta and class coccolithophyceae (or) prymnesiophyceae. *Emilinia huxleyi, Pleurochrysis carterae, Chrysotila carterae* are the most abundant and highly studied coccolithophore forming species in ocean waters 6. Coccoliths are complex elliptical objects ranging in few microns diameter 7, 8. Coccolithophores are a group of key marine phytoplanktons which exhibit characteristic external calcium carbonate (CaCO_3_) shells or plates called coccolith 9. Coccolithophores are well-studied for two major reasons: firstly, they are key contributors of global carbon cycles, marine ecosystem and ocean calcification and secondly because of its intricate structure 9. Coccolithophores strongly impact the biogeochemistry of ocean's surface through its photosynthesis and coccolith also release CO_2_ during its formation 10. Studies have also reported the role of coccolithophores in global sulfur cycling as they produce dimethyl sulfoniopropionate (DMSP) 9. Coccolithophores are naturally synthesized through a biomineralization process called coccolithogenesis, the light-dependent calcification process of the coccolith scales is found to be high during the exponential phase of its growth 11. The calcification/calcite construction is initiated in the Golgi complex. Firstly, the peripheral calcite crystals are centralized onto an organic base plate, which is produced from coccolith vesicle, which is derived from the Golgi complex 12. The calcite scales produced are exported on to the coccolith vesicles which add these new vesicles to the inner surface of the coccosphere. Thus, newly produced coccoliths are added underneath the older coccoliths 13. Based on the phytoplankton's life stage two types of coccoliths are produced (1) holococcolith (produced in haploid phase, ranges anywhere between hundreds to thousands of calcite scales) and (2) heterococcoliths (produced in diploid phase and are composed of less than hundred complex calcite crystals) 14-17.

*Pleurochrysis* genus phytoplanktons are popularly studied coccolithophore producing ocean water algae. These marine phytoplanktons are classified under Haptophyta (division), *Prymnesiophyceae* (class)*,* coccolithales (order) and *Pleurochrysidaceae* (family) 18. Popularly known *Pleurochrysis* genus algae are: *P. carterae*, *P. dentata*, *P. elongate*, *P. gayraliae, P. haptonemofera, P. placolithoides, P. pseudoroscoffensis, P. roscoffensis* and *P. scherffelii*. *P. carterae* is a highly studied phytoplankton from the *Pleurochrysis* genus. *P. carterae* is a photoautotrophic unicellular marine alga. Cells are mostly round or oval and contain two chloroplasts 19, 20. Like most photosynthetic organisms, *P. carterae* can synthesize organic material through photosynthesis, providing the necessary substances and energy for growth and reproduction. During its growth, *P. carterae* cells develop a scaly surface structure called coccolith, which is mainly composed of CaCO_3_ crystals synthesized via calcification. The reason why the coccolith exists is still not clear. However, some hypotheses reported on coccolithophores stated that a) it protects the cell; b) it reduces the effects of excessive light on growing algae; c) it might increase/ decrease its exposure to the light, thus playing a role in its photosynthesis; d) it increases the sedimentation rate of cells 21-23.

*P. carterae* was also found to contain high levels of lipids; the lipid content of *P. carterae* can reach 33% of dry weight 24. Previous studies have reported that it contains many valuable dietary fats such as ω - 3 Polyunsaturated Fatty Acids (PUFAs) 25, 18:3 ω-3, 18:4 ω-3, 18:5 ω-3, 20:4 ω-3, 20:5 ω-3 (Eicosapentaenoic Acid 26), 22:5 ω-3, and 22:6 ω-3 (Docosahexaenoic Acid DHA). EPA and DHA are highly valued compounds in food and pharmaceutical industries, especially EPA and DHA were effective against cardiovascular diseases 27. Recent studies have also confirmed that *P. carterae* has great potential as a biodiesel producer 24, 28, 29.

Excessive nutrients (N, P^3-^, K^+^) in ocean environments leads to eutrophication, which further leads to algal blooms 30. Algal blooms release many volatile chemical substances such as dimethyl sulfide (DMS), acrylic acid, etc. Algal blooms seriously effect the local environment, ecosystem, and marine economy 31. Takagi et al (2000) have reported that* Nannochloris sp* cultured with 0.9 mM concentration of KNO_3_ accumulated higher amounts of lipids compared to the *Nannochloris sp.* cultured with 2.0-9.9 mM concentrations of KNO_3_, respectively 32. Zhang et al (2004), has reported that *P. carterae* cultured with different concentrations of KNO_3_ ranging between 0.25, 0.5, and 0.75 mmol·L^-1^ had shown significant effects on its growth cycle 33. Our present study is focused on understanding the effects of KNO_3_ on CaCO_3_ accumulation, lipid, carbohydrate and protein contents of *Pleurochrysis dentata.* We have also extensively described the structural and morphological properties of *Pleurochrysis dentata* coccoliths using SEM microscopy. To the best our knowledge, this is the first report explaining the tentative novel mechanism involved in synthesis of CaCO_3_ and coccolith shell three-dimensionally.

## Materials and Methods

### Algal strain

*Pleurochrysis sp.* was kindly provided by Dr. Fan Lu, Hubei University of Technology, China.

### Preparation of f/2 medium

*Pleurochrysis sp.* was cultured using the standard f/2 medium (pH 8.0) composed of 225 mg L^-1^ NaNO_3_, 5.6 mg L^-1^ NaH_2_PO_4_·H_2_O, 1 ml L^1^ trace metal solution, and 0.5 ml L^-1^ vitamin solution (Guillard and Ryther 1962). The trace metal solution is composed of 315 mg FeCl_3_·6H_2_O, 436 mg Na_2_EDTA·2H_2_O, 9.8 mg CuSO_4_·5H_2_O, 6.3 mg Na_2_NoO_4_·2H_2_O, 22 mg ZnSO_4_·7H_2_O, 10 mg CoCl_2_·6H_2_O, and 180 mg MnCl_2_·4H_2_O dissolved in a final volume of 100 ml distilled water. The vitamin solution is composed of 20 mg thiamine HCl (vitamin B1), 10 mg biotin (vitamin H), and 10 mg cyanocobalamin (vitamin B12) dissolved in a final volume of 100 ml distilled water.

### Algal culturing and sample collection

The above prepared standard f/2 medium was supplemented with different concentrations of KNO_3_ (0.25, 0.5, 0.75, 1 mmol L^-1^), samples without KNO_3_ (0 mmol L^-1^) was considered as control. The starting algal cell density was 1.5×10^5^ cells per liter. The culture temperature was set at 26 °C with 16/8 hours light and dark photocycle maintained by cold white fluorescent light controller under 24 hours continuous circulation of air. From the above maintained algal cultures 200 ml of *P. dentata* cells were collected on 0, 2, 4, 6, 8, 10 days. The collected samples were centrifuged at 5,000 r·min^-1^ for 10 minutes. Thus, obtained pellets were washed twice with distilled water and later the samples were dried in oven at 105 °C until the weight remained stable.

### Calcium content measurement

The calcium content estimation analysis mainly contains two steps:

*(a) Sample decolorization:* The samples decolorization step was performed using 75% ethanol 34-36. The decolorization process was initiated by adding 1ml of 75% ethanol to 0.01 mg of the dry algae, obtained from oven drying. The tubes were transferred to a hot water bath set at a temperature of 80 ℃ and incubated for 30 minutes. After the incubation, samples were centrifuged at 12,000 r min^-1^ for 30 minutes, followed by discarding the supernatants. The above decolorization process was repeated until the supernatants remained colorless.

*(b) Calcium content measurement:* Above obtained decolorized samples were mixed with 1.5 ml of 2 N HCl and incubated in a water bath maintained at 80 °C temperature for 30 minutes to remove CO_2_. The CaCl_2_ supernatants were collected by centrifuging at 12,000 r·min^-1^ for 3 minutes. The calcium ion content in the supernatant was measured using the ethylenediaminetetraacetic acid (EDTA) complexmetric titration method 37. The Calcon^TM^ indicator obtained from Millipore Sigma® was used to determine the formation of final colored Calcon-carboxylic acid complex. The reaction mixtures were constantly monitored for the color change from red to blue (remains for at least 30 seconds).

The calcium ion content (mg L^-1^) was calculated using the following equation:


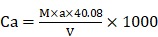


M: EDTA standard solution molar concentration.

a: The volume (ml) of EDTA standard solution is consumed during the titration.

V: Sample volume (ml).

The atomic weight of calcium: 40.08.

The calcium ion content in the sample is calculated using the standard curve.

### Total lipid measurement

Total lipid contents of *P. dentata* samples were determined using the standard chloroform-methanol extraction method 38. About 0.01 g of oven dried *P. dentata* was transferred to a 1.5 ml centrifuge tube. The reaction was initiated by adding 395 µl of chloroform, 790 µl methanol, and 316 μl of deionized water at a ratio of 1:2:0.8 (V: V: V), respectively. The reaction mixtures were then mixed by vortex for 3 to 5 minutes, followed by centrifugation at 12,000 r min^-1^ for 3 minutes; supernatants were further collected into a glass tube. The above reaction step was repeated for three times. The final reaction mixture contains the above collected supernatant and 395 µl of chloroform, 395 µl methanol, and 316 µl of deionized water added in a ratio of 1: 1: 0.8 (V: V: V), respectively. The reaction mixtures are mixed well and stored at room temperature until it separates into layers. The bottom layer was then transferred to a pre-weighed tube, record it as M1 (g) later the tube is placed in the fume hood overnight. The tube was later dried in oven at 105 °C until the weight remained constant, weigh this oven dried samples and record it as M2 (g).

The lipid content was calculated using following equation:


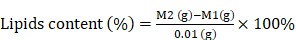


### Carbohydrate content measurement

The phenol-sulfuric acid method was used for determining the carbohydrate content of *P. dentata* 39. The 0.01 g dried algal powder samples were transferred into 15 ml centrifuge tube containing 2 ml distilled water and 600 µl of concentrated HCl. The reaction tubes were incubated in a water bath at 100 °C, after 3 hours of incubation; the samples were cooled to room temperature and filtered using Whatman No.1 filter paper. The above-obtained residues were washed using distilled water for three times. The washed residues were re-suspended using 10 ml of distilled water and centrifuged at 12000 r·min^-1^ for 2 minutes, these supernatants were collected for total sugar determination. The final reaction mixture consists of 1 ml of 5% phenol solution, 2 ml supernatant and 5 ml of concentrated sulfuric acid solution. The reactions was initiated by adding 1 ml of 5% phenol solution to 2 ml supernatant and once the reaction mixture is well mixed, quickly add 5 ml of concentrated sulfuric acid solution and store at room temperature. After 10 minutes the samples were vortexes to attain evenly mixed solutions, the reaction is initiated by incubating it in water bath with temperature set at 40 °C. After 30 minutes, samples were cooled down by incubating the tubes in cold-water bath for 5 minutes. The absorbance was measured at 490 nm using distilled water as a blank in an Epoch™ Microplate Spectrophotometer (BioTek®, Inc., headquartered in Winooski, VT, USA).

### Determination of protein content

Protein content of *P. dentata* were determined using SK3041-1000 Assays Better Bradford Protein assay kit, by following the manufacturer provided instructions (Bio Basic Canada Inc., Markham, ON, CA). The 0.01 g oven dried *P. dentata* was transferred to 2 ml centrifuge tube placed on crushed ice; add 1 ml of 0.15 M NaCl solution. Later the samples were sonicated at 40 kHz by placing the 2 ml centrifugation tube on a beaker containing crushed ice. After 10 minutes, the tubes were centrifuged at 12,000 r min^-1^ for 2 minutes. The supernatants were discarded, and pellets were washed for three times using distilled water. Finally, 0.15 M NaCl solution was added to the pellet, followed by centrifuging at 12,000 r min^-1^ for 5 minutes to extract the protein. The supernatants containing proteins were first stored in a bench top cooler and later subjected to protein measurement. The reaction was initiated by adding 150 µl supernatant and 150 µl Bradford reagent to 96-well plate. The reaction mixture was stored at room temperature after 15 minutes the samples were analyzed using Epoch™ Microplate Spectrophotometer (BioTek®, Inc., headquartered in Winooski, VT, USA) at 595 nm. The protein contents of *P. dentata* were determined using bovine serum albumin standard at concentrations ranging between 0-30 mg mL^-1^, respectively.

### Sample fixation and SEM Analysis

The *P. dentata* cells were fixed using 2.5% to 3% concentration of glutaraldehyde with the pH ranging between 6.8 and 7.4; the pH was maintained using phosphate buffer solution 40. The *P. dentata* cells were placed in the glutaraldehyde fixative solution for about 30 minutes to ensure complete infiltration of the glutaraldehyde solution into the *P. dentata* cells*.* The above fixed *P. dentata* cells were subjected to gradient dehydration using different concentrations of ethanol (50%, 60%, 70%, 80%, 90%, and 100%). All the subjected *P. dentata* samples were sequentially soaked in each concentration starting from 50% to 100% for at least 15 minutes, later these samples were preserved in -80 °C freezers until its use. The *P. dentata* samples were removed from the -80 °C freezer and placed in a freeze dryer (LABCONCO freezone 12, Kansas City, MO, USA) for 24 hours. The samples were then sputtered with gold or carbon and later observed using HITACHI SU-70 Scanning Electron Microscope for imaging, respectively.

### Fourier-Transform Infrared Spectroscopy (FTIR)

Accuracy of all the above obtained experimental results for carbohydrate, protein and lipid contents was verified using Fourier-Transform Infrared Spectroscopy (FTIR) BRUKER® TENSOR 37. The characteristic absorption band of lipids was found in between the range of 3000-2800 cm^-1^ (representing the C-H stretching vibration in acyl chains) which was used to characterize the change in lipid content of *Pleurochrysis dentata* test samples. Similarly, the peptide linkages of proteins were analyzed based on the C=O stretching and N-H bending vibrations by observing at 1650 cm^-1^ (amide I) and 1540 cm^-1^ (amide II) bands, respectively. Thus, changes in protein content correspond to its absorption strength. Finally, the changes in carbohydrates were analyzed between the range of 950-1200 cm^-1^ absorption bands for analyzing the C-O-C vibration, and the resulting absorption strength directly corresponds to its changes in total carbohydrate contents 41-43.

### 18S rRNA Sequencing of the *P. dentata.*

To identify the *P. dentata* strain, we have performed 18S rRNA sequencing based on the protocol reported by 44. The polyvinylpyrrolidone (PVP-40) with an average molecular weight of 40 kDa and BSA fraction V were purchased from Millipore Sigma®. The 18S rRNA isolation was performed using buffer A and B. Buffer A must be freshly prepared using 10 M NaOH and 20% Tween 20 stock solutions just before use, and 100 mM Tris-HCl obtained from Millipore Sigma® is used as Buffer B solution respectively. Firstly, the *P. dentata* culture sample (approximately 30 mm^2^) was transferred to 96-well plate. To the above 96-well plates add 50 µL buffer A solution and incubate the samples at 95 °C for 10 minutes. After 10 minutes add 50 µL buffer B solution and mix at moderate speed. Later aliquot PCR mixture to 96-well plates at 20 µL/well (1 µL subject DNA sample , 1.5 mM MgCl_2_, 0.2 mM each dATP, dCTP, dGTP, and dTTP, 0.25 µM each forward and reverse primer, 0.1% BSA (w/v), 1% PVP (w/v), and 0.5 U HotStart Taq DNA polymerase). Transfer approximately 1 µL of DNA sample from the DNA plates to PCR plate respectively. The standard 18S rRNA forward: GAAACTGCGAATGGCTCATT and reverse: CCTTCTGCAGGTTCACCTAC primers were used. The polymerization was performed by the Bio-Rad® PCR machine using the standard conditions. The polymerized 18S rRNA gene products obtained were characterized using 1% agarose gel electrophoresis. Finally, the above characterized 18S rRNA product was retrieved using the Gel/PCR DNA fragment extraction kit. The gene product obtained from the gel extraction kit is further sent to the sequencing company. The 18S rRNA sequence of *P. dentata* strain is reported in the supplementary information, respectively. The 18S rRNA sequence of *Pleurochrysis dentata* LU1 strain was deposited in NCBI GenBank under the accession number: MN186658.

We have also validated the 18S rRNA sequence results by isolating the mRNA sequences of *P. dentata* and converted it to cDNA, protocol used, and the results obtained from this method was reported in supplementary information respectively (Sequence-S2, Table-S7 and Figure-S2).

### Sequence and Phylogenetic Analysis

The 18S rRNA sequences of *P. dentata* was first subjected to standard nucleotide BLAST analysis (using the default settings) against the nucleotide collection (nr/nt) database and the optimized for the highly similar sequences (mega blast), respectively. Results obtained from the BLAST analysis were reported in Supplementary information (Table-S1). We have retrieved the top 19 18S rRNA sequences exhibiting highest similarity. Thus, obtained sequences were analyzed using MEGA-X software version 10.0.5, by first aligning the sequences using ClustalW and these aligned sequences were further subjected to phylogenetic analysis. The evolutionary history was inferred using the Neighbor-Joining method 45. The bootstrap consensus tree inferred from 500 replicates 46 was taken to represent the evolutionary history of the taxa analyzed 46. Branches corresponding to partitions reproduced in less than 50% bootstrap replicates were collapsed. The percentage of replicate trees in which the associated taxa clustered together in the bootstrap test (500 replicates) are shown next to the branches 46. The evolutionary distances were computed using the Maximum Composite Likelihood method 47 and are in the units of the number of base substitutions per site. This analysis involved 20 nucleotide sequences. All ambiguous positions were removed for each sequence pair (pairwise deletion option). Evolutionary analyses were conducted in MEGA-X v10.0.5 48. The obtained sequence was deposited in NCBI GenBank the under-accession number MN186658.

### Statistical Analysis

All the experiments were conducted in triplicates. The results obtained from all the triplicates were subjected to statistical analysis using one-way ANOVA function of the IBM SPSS^®^ software. We have selected “Duncan's multiple-range test” as the comparison method with the level of significance (P-value) set at 0.05. We have individually reported the statistical significance results for CaCO_3_, total carbohydrate, lipid, protein contents in supplementary information file.

## Results

### Identification and Phylogenetic Analysis of *Pleurochrysis dentata*

**Figure 1:**
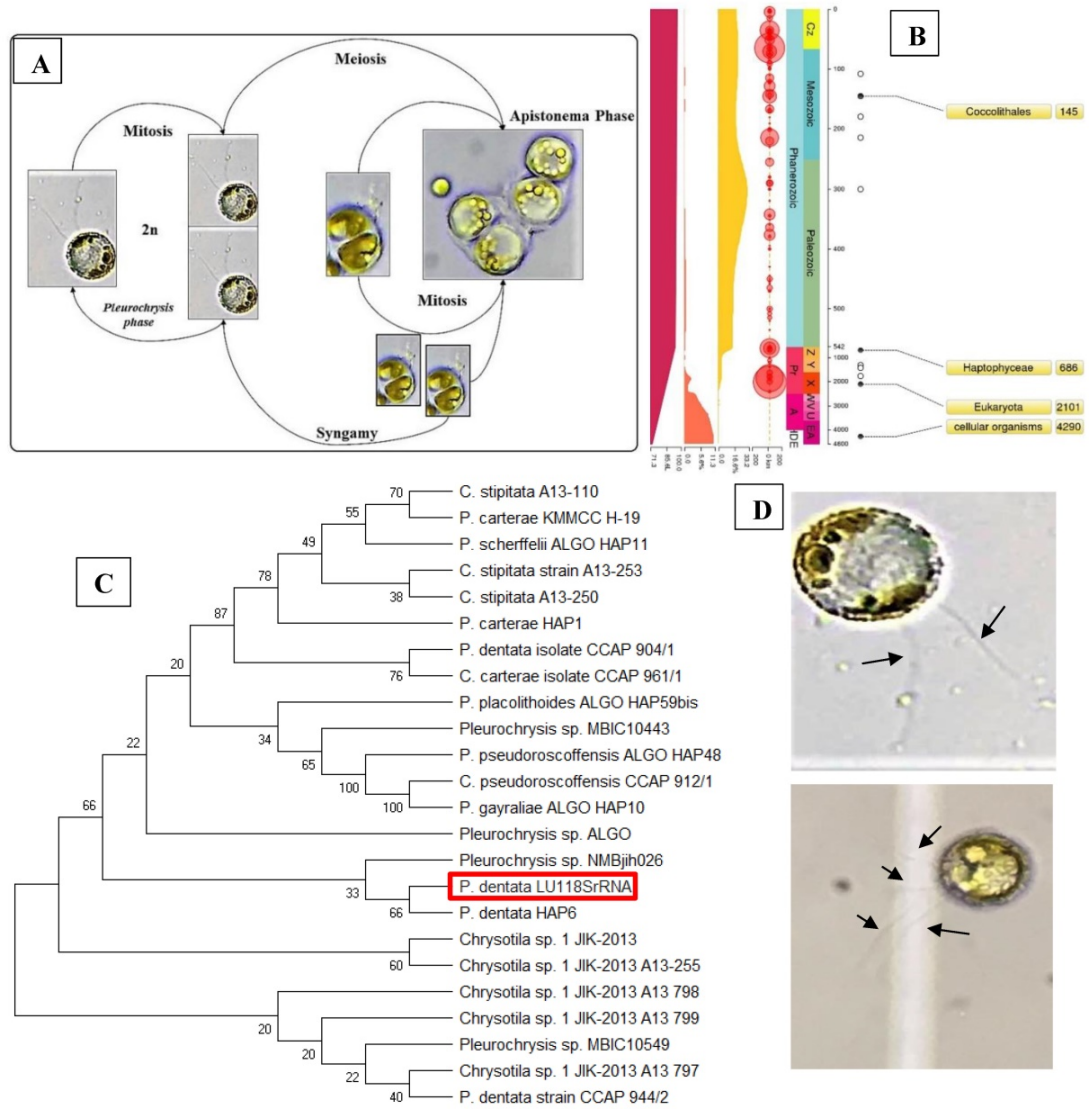
** (A)** Compound microscopy images of *P. dentata* life cycle showing alternation of generations; **(B)** TimeTree analysis of *Pleurochrysis* genus; **(C)** phylogenetic analysis of *Pleurochrysis sp.* 18S rRNA; **(D)** compound microscopic images showing fully developed *P. dentata* with two and four flagella.

The *Pleurochrysis dentata* strain was cultured using the standard f/2 algae culture medium for 10-days in long-jars by providing standard algal growth conditions. *Pleurochrysis dentata* are golden yellow-green algae with a central nucleus, anterior Golgi complex, two plastids, thylakoids and pyrenoids (sub-cellular microcompartments present in chloroplasts). *Pleurochrysis dentata* are kinetic and they possess all the necessary apparatus required for its motility including 2 flagella and a haptonema. *Pleurochrysis dentata* cells are surrounded by three membranes a) proximal columnar material next to the cell membrane, b) multiple layers of unmineralized scales and c) distal layer of calcified interlocking coccolith scales. We have observed the life cycle of *Pleurochrysis dentata* using compound microscope. Previous studies have reported that *P. dentata* exhibits an alternation between haploid and diploid generations with an alternation of a non-motile stage called as Apistonema stage containing one or more motile forms (Figure 1A). We performed a preliminary study to understand the evolution of *Pleurochrysis* genus using TimeTree of life web-database. Results from this analysis showed that *Pleurochrysis* genus has evolved during Mesozoic period with an estimated divergence time of 145 Mya (Figure 1B). To identify the *P. dentata* phylogeny we have isolated the DNA sequence coding for *P. dentata* and using 18S rRNA primers we have polymerized and later sequenced the obtained PCR products. Thus, obtained 18S rRNA sequences were analyzed for its phylogeny using NCBI-BLAST web-database and MEGA software version 10.0.5. Results obtained from BLAST analysis showed that the forward and reverse primer of *P. dentata* 18S rRNA exhibited highest similarity with *Pleurochrysis dentata* HAP6 and *Chrysotila sp.* 1JIK-2013 strain A13801, respectively [18S rRNA sequence in Supplementary information] (Table-S1) (Figure 1C). We have also sequenced the 18S rRNA PCR products obtained from *P. dentata* cDNA (*P. dentata* mRNA was isolated and converted to cDNA). The 18S rRNA sequence obtained from this method have also exhibited highest similarity towards *P. dentata* HAP6 and *Chrysotila sp.* 1JIK-2013 strain A13801 respectively (Table S7, Figure S2).

### Novel *P. dentata* calcium carbonate shell structure

Current research showed that *P. dentata* is covered by oval shaped CaCO_3_ shells known as the coccolith or double disc of calcite 49. The coccoliths are composed of two parts, the first part is called the radial (R unit), and second part is called the vertical (V unit) 50. Earlier studies have reported that formation, structure and size of coccoliths are controlled by complex acidic polysaccharides 51-53. Morphological and crystallographic studies of coccolithophore mineralization and structural orientation of V and R units during different stages of *Pleurochrysis carterae* life cycle were first reported by Marsh (1999) 54 (Figure-S1A, B, C). According to Kazuko, et al (2011), the morphological and crystallographic alignment studies of *Pleurochrysis carterae* coccoliths at various life stages has revealed that V and R units of immature coccoliths are not fixed on the organic base plate, once matured the V and R units interlock with each other resulting in an anvil-shape 55.

**Figure 2:**
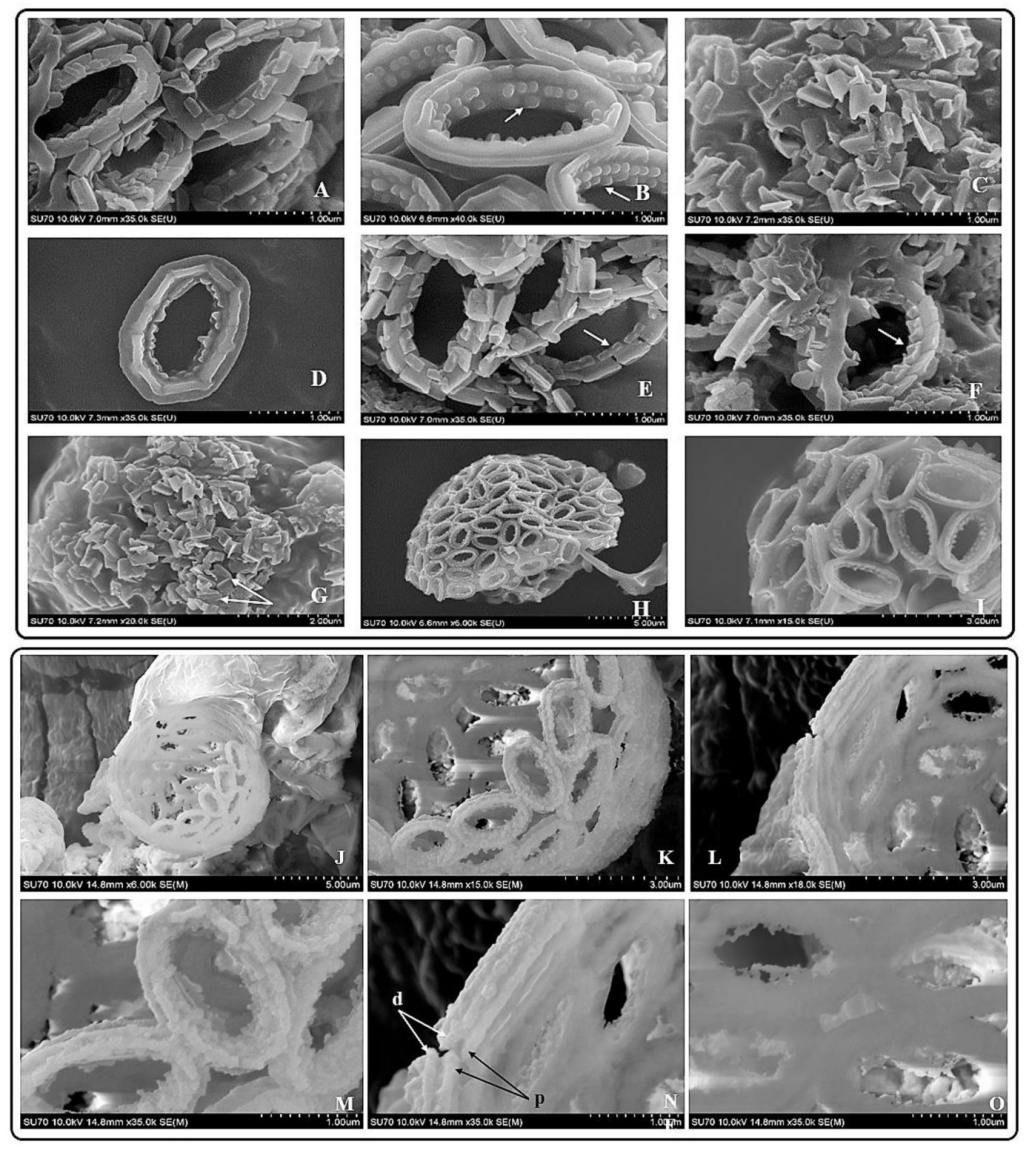
** SEM pictures of *P. dentata* coccolith.** (i) **(A)** Loose structure of coccolith. **(B, E, F)** The arrow points to “doornail structure”. **(C)** Cracked coccolith. **(D)** Single coccolith.** (G)** The arrow points to "R unit". **(H, I)** *P. dentata* wrapped by coccolith. (ii) Support Structure of CaCO_3_ cell wall. **(J)** an empty shell without protoplasts. **(K)** Close range view. **(L)** Adjacent two coccolith side views. **(M)** Top view of two adjacent coccoliths. **(N)** Enlarged side view of two adjacent coccoliths. **(O)** Rear view of several adjacent coccoliths. **Arrowhead *d*:** distal-shield. **Arrowhead *p*:** proximal-shield.

**Figure 3:**
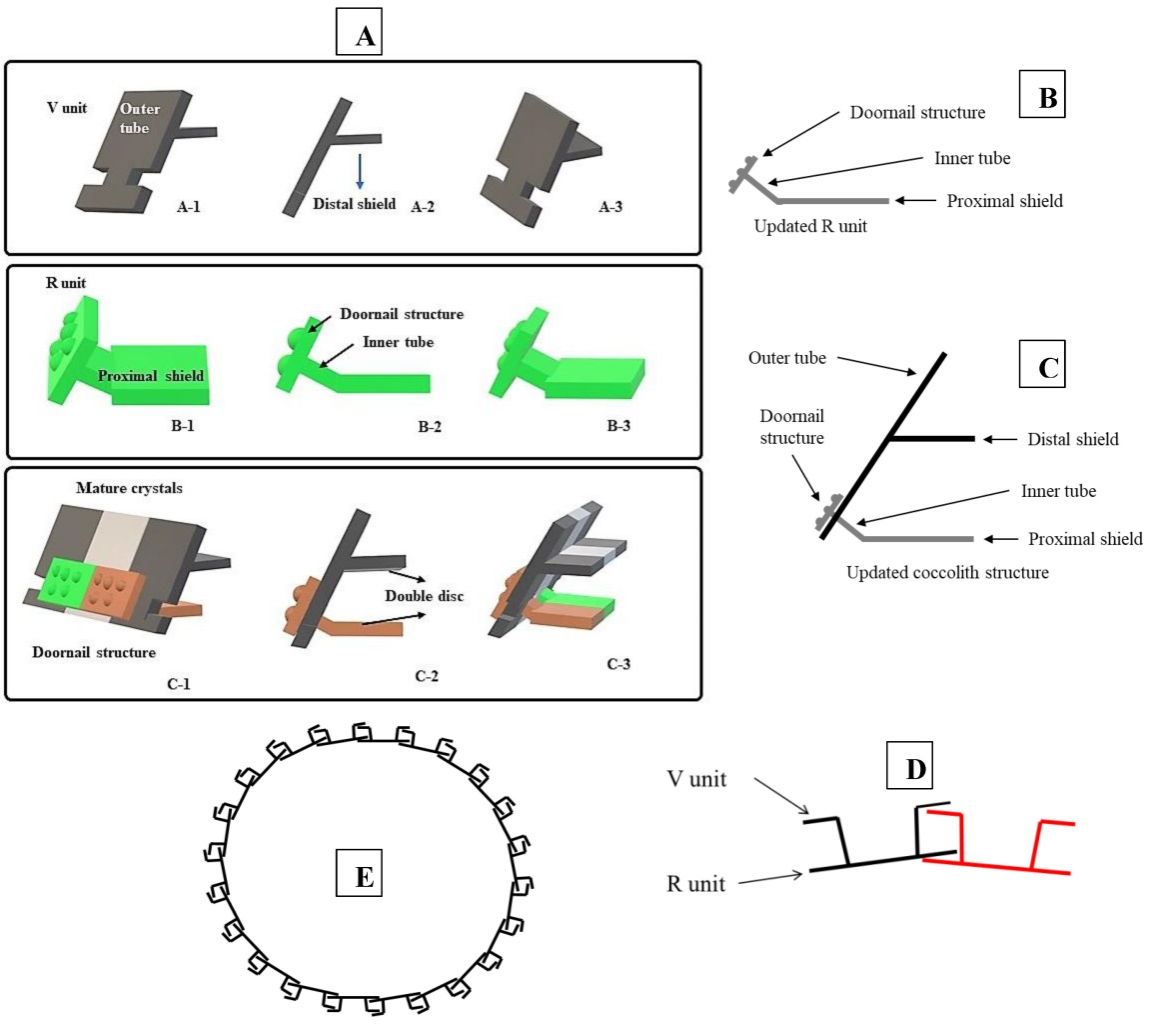
**Tentative CaCO_3_ shell 3D structure. (A) A-1**: Outer tube of V unit. **A-2**: Side view of the V unit and the distal-shield. **A-3**: 3D graph of V unit. **B-1**: Proximal-shield of R unit. **B-2:** Side view of the R unit includes inner tube and doornail structure. **B-3**: Updated 3D graph of R unit. **C-1**: Front view of the combination of V unit and R units. **C-2:** Side view of combination structure of V unit and R unit. **C-3**: Rear view of combination structure of V unit and R unit; **(B)** Simplify graph of side view of R unit; **(C)** Simplify graph of side view of combination structure, **(D)** A tentative model showing biological arrangement of two coccoliths; **(E)** A tentative model showing extracellular coccolith support structure (or) support structure of CaCO_3_ cell wall.

Results obtained from the morphological studies of *P. dentata* coccolith have revealed that “on top of the inner tube, there is a baffle structure with circular convex (Figure 2 and Figure 3). Moreover, based on our observations, this structure is parallel with the outer tube of the V unit (Figure 2A, E, F). We named this baffle structure as the “doornail structure”. The complete coccolith structure is shown in our experiments (Figure 2B, D), and from these images, it can be noticed that the structure of coccolith has started to disperse, but the presence of doornail structure keeps overall structure intact together (Figure 2A and E). The doornail structure ensures that the coccolith has stronger stability and is not easily collapsed. Using the results obtained from morphological studies on *Pleurochrysis carterae* (Figure S1B) 54 and based on the results from our study (Figure 2B,C,E,F,G), we have proposed a novel 3D model of coccolith formation (Figure 3A), side view of the model R unit and interlocking structure (Figure 3B and 3C). We have successfully reported the complete coccolith shell; Figure 2J captures the fully developed coccolith* P. dentata* without the protoplast. The distal-shield (V-unit) and proximal-shield (R unit) of the *P. dentata* coccolith were reported in Figure 2N and the units were represented with white and black arrows, respectively. These images clearly report that the distal-shield and proximal-shield are crossed together, and this cross structure is responsible for the entire propped up outer shell. The crossing over of the distal and proximal shields ensures that the outer shell does not spread. The alignments of the distal and proximal shields in coccolith of *P. dentata* were shown in Figure 3D. From the SEM images (Figure 2K, M, and O) we can observe that edges of coccolith are closed together. Based on all the above obtained results, we have reported a simple graphical structure of the entire shell (Figure 3E). The double disc edges of each coccolith crossed together serves as a support and ensures the integrity of the entire coccolith structure. As single-cell phytoplankton, this scaly coccolith shell has attained great flexibility in its evolution. This cross-supporting structure can be stretched and not damaged by the expansion of the protoplasts during its cell division (Figure-S1A, B, C).

### The Effect of KNO_3_ on *P. dentata* CaCO_3_ accumulation

**Figure 4:**
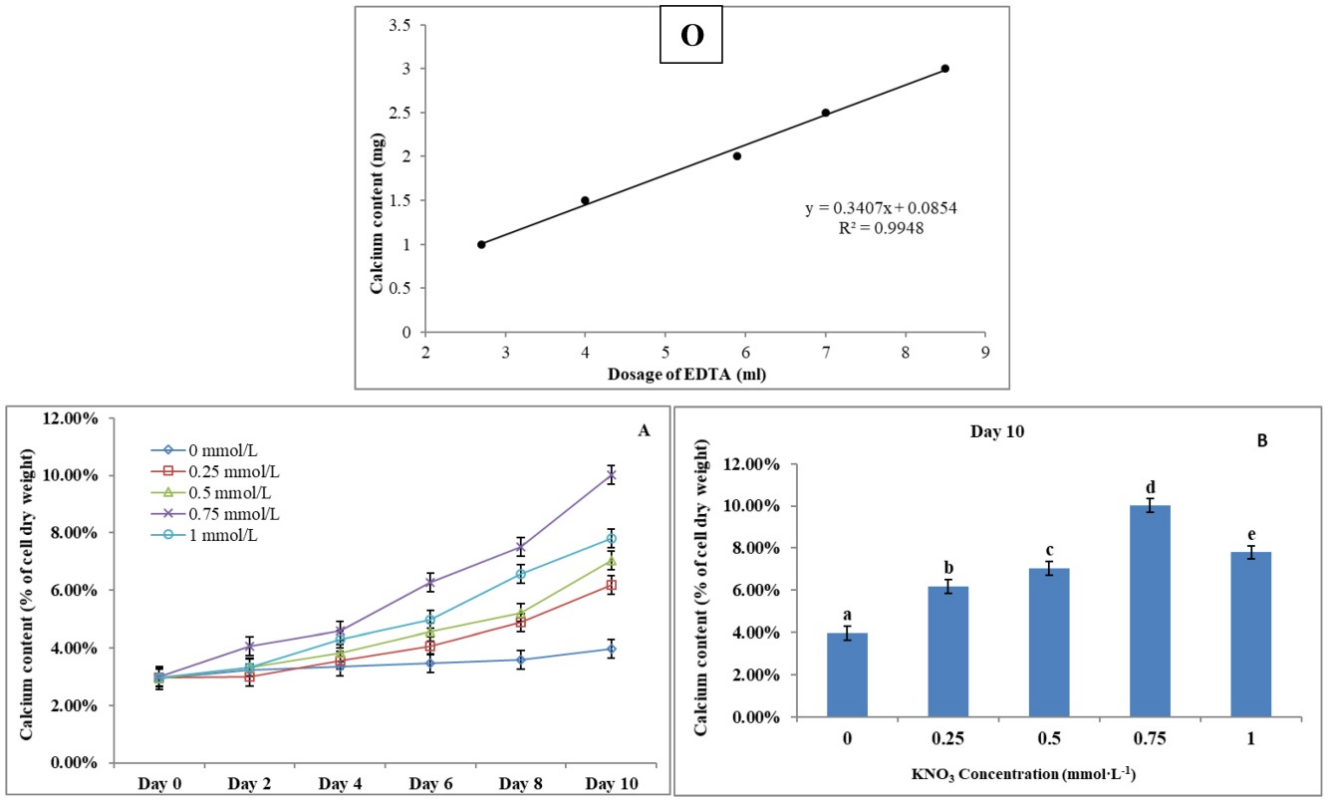
** (O)** Calcium ion content standard curve.** (A)** Ten days' calcium content trend of *P. dentata* growing in f/2 medium containing different concentrations of KNO_3_ (0, 0.25, 0.5, 0.75, and 1 mmol L^-1^);** (B)** Calcium content of *P. dentata* in different KNO_3_ concentration culture mediums on day 10. [Note: a b c d e alphabets indicates the significant difference (P<0.05) among the test conditions, given by Duncan's multiple range test].

**Figure 5:**
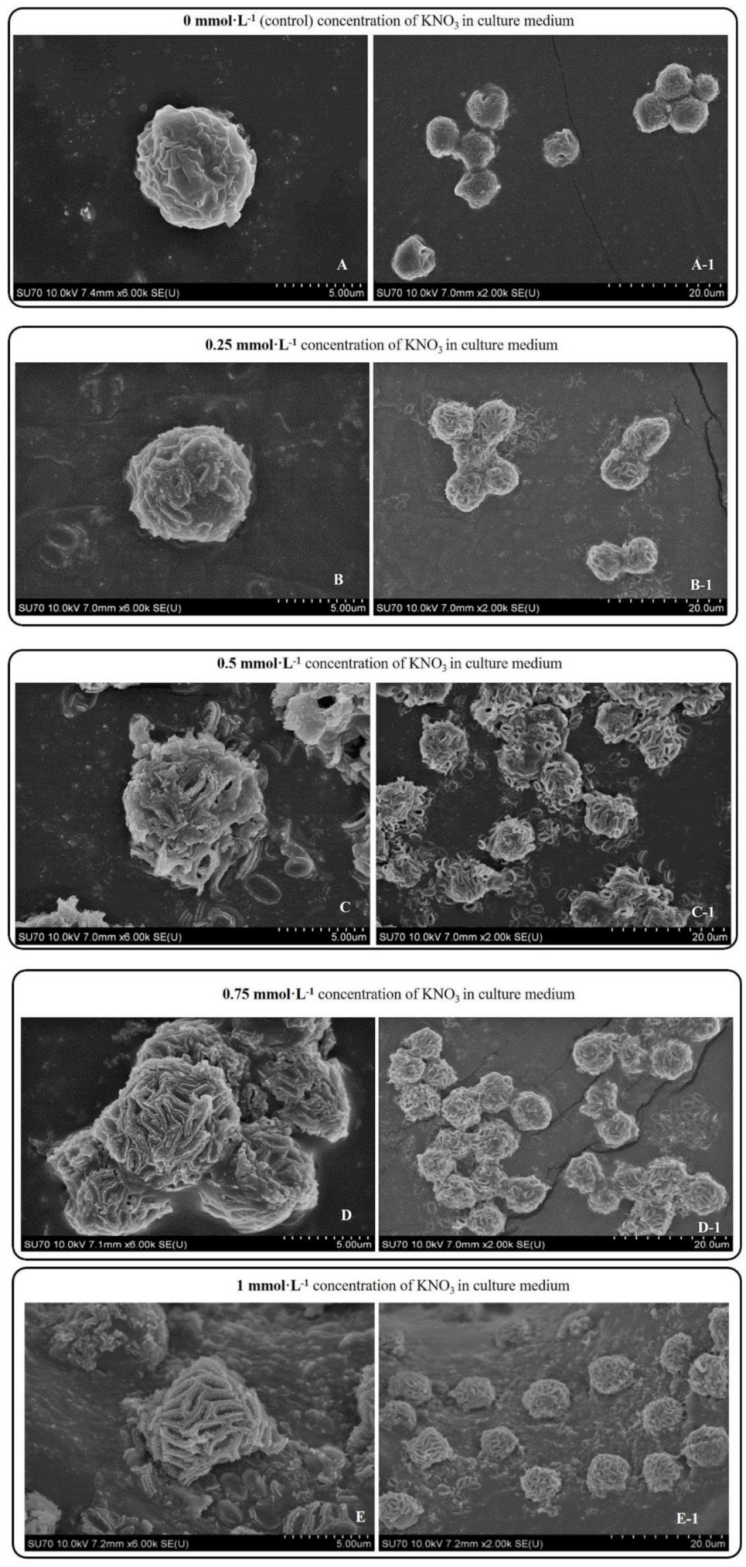
Day 10 SEM images *P. dentata* cultured at 0 mmol L^-1^ **(A)**, 0.25 mmol L^-1^ **(B)**, 0.50 mmol L^-1^ **(C)**, 0.75 mmol L^-1^ **(D)**, 1 mmol L^-1^ **(E)** KNO_3_ concentrations supplemented with f/2 medium.

*P. dentata* cultured under increasing concentrations of KNO_3_ starting from 0.25 to 1 mmol L^-1^ with 0 mmol L^-1^ as control. We have constructed a standard curve using the CaCl_2_ concentration (Figure 4O). We have observed a gradual increase in calcium ion concentrations of *P. dentata* cells from 0 to 10 days. Interestingly, 10^th^ day cultures exhibited maximum calcium content compared to other samples under all the tested KNO_3_ concentrations (Figure 4A). The day 10 samples of *P. dentata* cultured using 0.75 mmol L^-1^ KNO_3_ concentration accumulated highest calcium carbonate with 10.01% of dry weight (Figure 4B). We have also performed SEM image analysis to observe the changes in CaCO_3_ content on the surface of *P. dentata* (Figure 5). From the SEM image results, we have observed that KNO_3_ exhibited a significant effect on CaCO_3_ synthesis. Thus, higher the concentration of KNO_3_, higher the rate of calcium carbonate accumulation in *P. dentata* Cells. *P. dentata* cultured using 0.75 mmol L^-1^ KNO_3_ concentration exhibited the largest cell size and highest CaCO_3_ accumulation (Figure 5D). Compared to other concentrations, *P. dentata* cultured at 1 mmol L^-1^ concentration of KNO_3_ were mature and the cells were found to grow individually (Figure 5 A-1 to E-1). However, the cell size and CaCO_3_ contents of *P. dentata* grown in 1 mmol L^-1^ were lower when compared with the cells cultured at 0.75 mmol L^-1^. The SEM images especially Figure 5 A-1 to E-1 and Figure 5 D-1 exhibited highest cell counts. Although the cell counts of C-1 were more than cell counts in E-1, interestingly the surface of E-1 cells exhibited higher coccoliths. Thus, *P. dentata* accumulated higher coccolith/CaCO_3_ shells at 0.75 mmol L^-1^ of KNO_3_ concentration and at 1 mmol L^-1^ KNO_3_ concentration could promote *P. dentata* cell division rate (Table-S2).

### The Effect of KNO_3_ on *P. dentata* lipid accumulation

**Figure 6:**
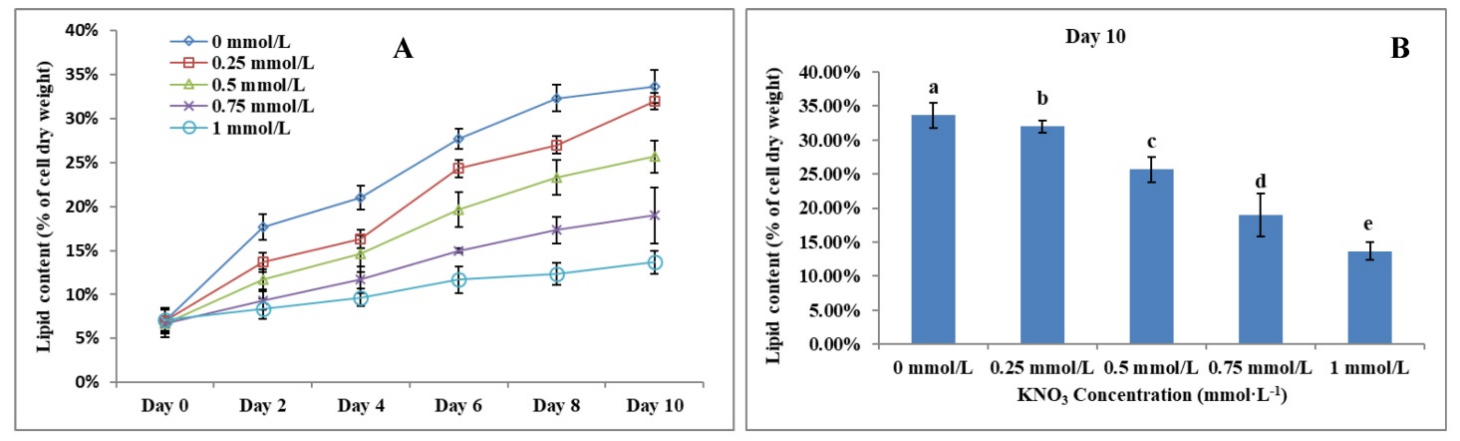
** (A)** Ten days' lipid content trend of *P. dentata* growing in f/2 medium containing different concentrations of KNO_3_ (0, 0.25, 0.5, 0.75, and 1 mmol L^-1^); **(B)** Lipid content of *P. dentata* in different KNO_3_ concentration culture mediums on day 10. [Note: a b c d e alphabets indicates the significant difference (P<0.05) among the test conditions, given by Duncan's multiple range test].

Lipid accumulation in KNO_3_ cultured *P. dentata* cells exhibited a gradual increase from day 0 to day 10 (Figure 6A, 6B). *P. dentata* accumulated highest lipid when it was cultured in f/2 growth medium without additional KNO_3_ (control or 0 mmol L^-1^) on day 10 (Figure 6B). Results obtained in this study have shown that, increasing concentrations of KNO_3_ has a negative effect on *P. dentata* lipid accumulation. Thus, with the increasing KNO_3_ concentration from (0.25 to 1 mmol L^-1^), we have observed gradual reduction in lipid accumulation. *P. dentata* cells cultured at 1 mmol L^-1^ concentration of KNO_3_ has accumulated lower amounts of lipid (lipid content reaching up to 13.67%) in day 10 cultures. On the other hand, *P. dentata* cells cultured with f/2 medium containing 0 mmol L^-1^ of KNO_3_ (control), accumulated lipid up to 33% of its dry weight in day 10 cultures 56, 57 (Table-S3).

### The Effect of KNO_3_ on total carbohydrate content of *P. dentata*

**Figure 7:**
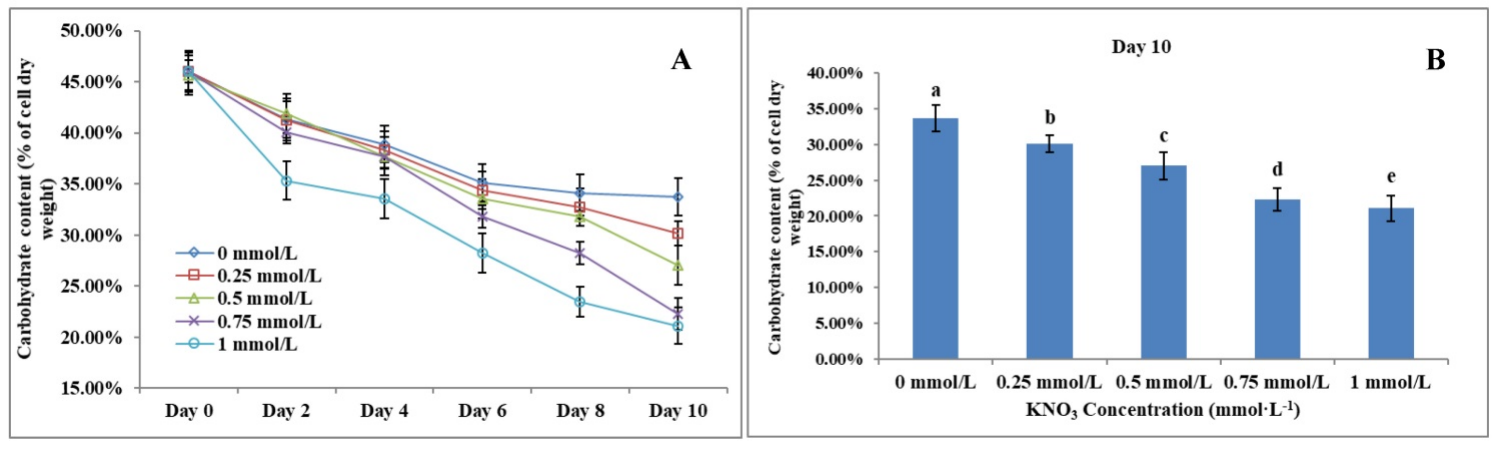
** (A)** Ten days' carbohydrate content trend of *P. dentata* growing in f/2 medium containing different concentrations of KNO_3_ (0, 0.25, 0.5, 0.75, and 1 mmol L^-1^);** (B)** Carbohydrate content of *P. dentata* in different KNO_3_ concentration culture mediums on day 10. [Note: a b c d e alphabets indicates the significant difference (P<0.05) among the test conditions, given by Duncan's multiple range test].

Carbohydrate content in microalgae generally ranges between 5 to 23% of its dry weight 58. Results obtained from the total carbohydrate content analysis showed that increasing KNO_3_ concentration exhibited a negative effect on carbohydrate accumulation in *P. dentata* (Figure 7A). The total carbohydrate contents of *P. dentata* were found to decline gradually with the increasing KNO_3_ concentration in the growth medium. Lowest carbohydrate content was recorded for growth cultures containing 1 mmol L^-1^ concentration of KNO_3_, whereas *P. dentata* cultured with 0 to 0.25 mmol L^-1^ concentration of KNO_3_, exhibited higher total carbohydrate content by containing 30% of its dry weight (Figure 7B). The carbohydrate content of the control group (0 mmol L^-1^) on day 10 reached a dry weight of 33.70%. Finally, the carbohydrate content of *P. dentata* reduced to 21.08% of its dry weight on its 10^th^ day, when cultured with 1 mmol L^-1^ KNO3 concentration. Thus, the total carbohydrate content decreased by 12.62% compared to the control group (Figure 7B) (Table-S4).

### The Effect of KNO_3_ on total protein content of *P. dentata*

**Figure 8:**
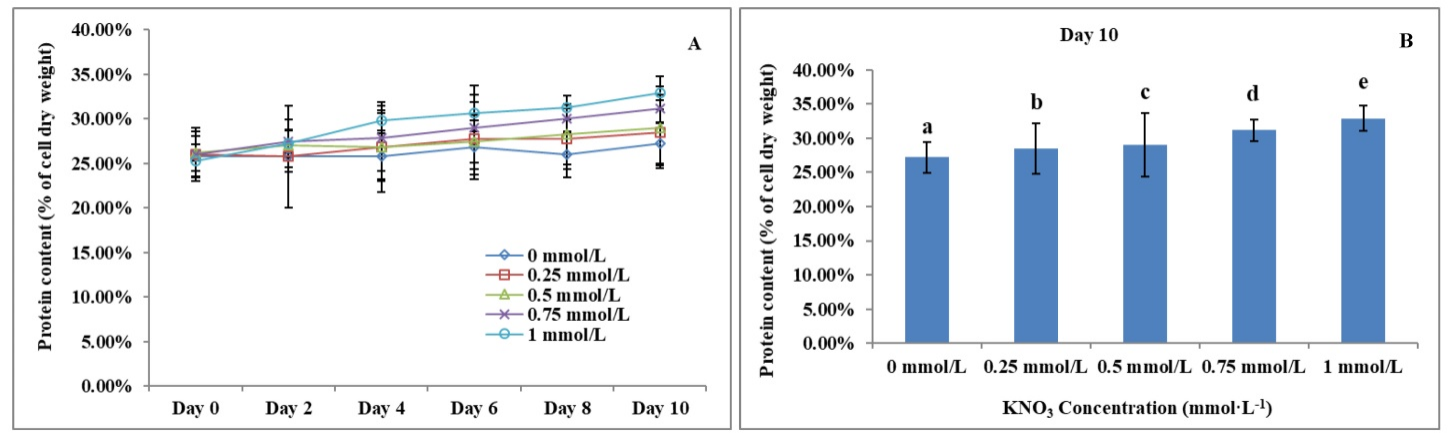
** (A)**Ten days' protein content trend of *P. dentata* growing in f/2 medium containing different concentrations of KNO_3_ (0, 0.25, 0.5, 0.75, and 1 mmol L^-1^); **(B)** Protein content of *P. dentata* in different KNO_3_ concentration culture mediums on day 10. [Note: a b c d e alphabets indicates the significant difference (P<0.05) among the test conditions, given by Duncan's multiple range test].

Previous studies have reported that the total algal protein dry weight percentage ranges between 3.19 to 78.10% 59-62. Studies have also reported that the total protein content of *P. dentata* accounts for 28.8% of its dry weight when cultured in Eppley medium 63. The total protein contents of *P. dentata* cultured under different concentrations of KNO_3_ for 10-days' time period were followed to show an increasing pattern from 0 to 1 mmol L^-1^ (Figure 8A). The 10^th^ day cultures of *P. dentata* containing 1 mmol L^-1^ of KNO_3_ concentration exhibited the highest total protein content, with 32.87% of its dry weight (Figure 8B), whereas the cultures containing 0 to 0.5 mmol L^-1^ concentration of KNO_3_ exhibited slightly lower total protein content (with 30% of its dry weight). The total protein content of the control group on its 10^th^ day accounted for 27.21% of its dry weight, which is 5.66% lower than the highest protein content (Figure 8B) (Table-S5).

### Fourier-transform infrared spectroscopy (FTIR)

**Figure 9:**
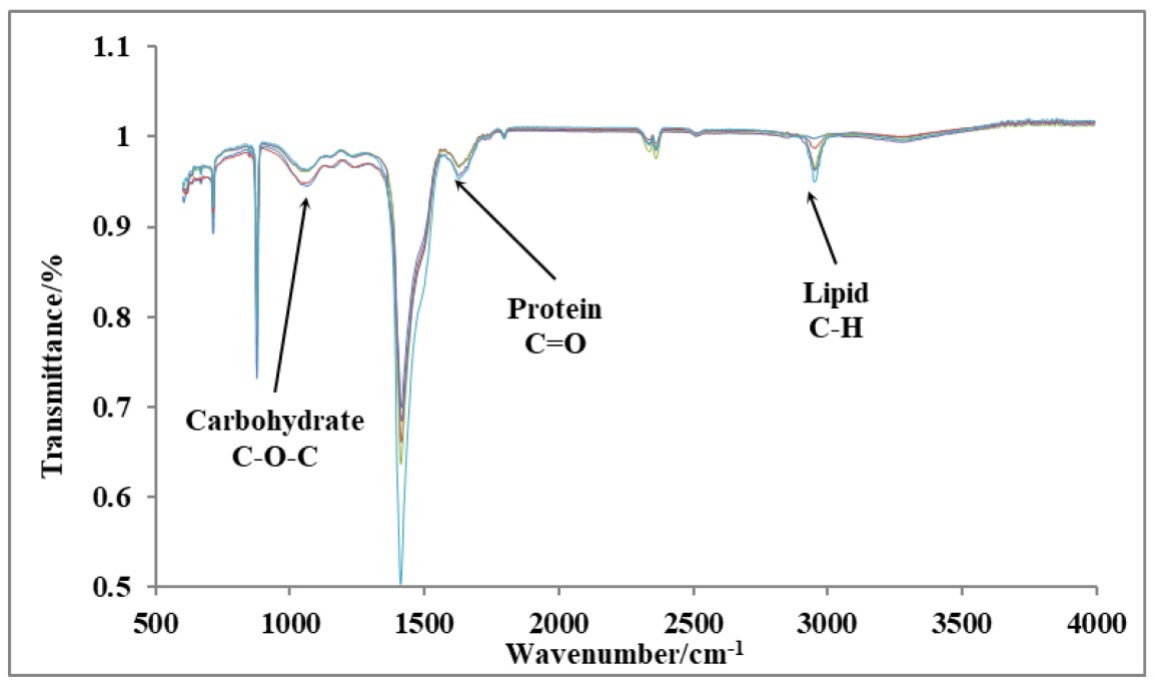
FTIR results of day 10 cultures *P. dentata* cultures showing the light transmittance on y-axis and wavelength on x-axis.

We have performed FTIR analysis on 10^th^ day cultures of *P. dentata* to verify the results obtained from our preliminary tests on the total lipid, protein, and carbohydrate contents using (Figure 9). The peaks indicated by three arrows represent carbohydrates (1072 cm^-1^), proteins (1649 cm^-1^), and lipids (2953 cm^-1^), respectively. The FTIR results are sensitive and from the graph shown (Figure 9) we can measure changes in the product type, and the observed transmittance is negatively related to the product content 41-43. The light transmittance results for the three products were shown in Table 1. These results show that the light transmittances for the day 10; 1 mmol L^-1^ KNO3 culture samples to be 99.81% for lipid samples, 95.98% for protein and 96.42% for carbohydrates, respectively (it exhibits similar trend as reported in Figure 4B). We have observed a decreasing pattern of light transmittance down the concentrations. The samples analyzed from the culture mediums without potassium nitrate exhibited a light transmittance of 97.24% for proteins (it exhibits a similar trend as reported in Figure 6B). The light transmittance for culture mediums without potassium nitrate for carbohydrate sample was 94.64%.

## Discussion

Earlier studies have explained about the single coccolith structure of *P. carterae* 49, 64, which mainly proposed that R unit contains two parts, the inner tube and the proximal-shield. We have observed a novel structure during our preliminary experiments studying the surface calcification of *P. dentata* (Figure 2 and 3). This structure is connected to the inner tube and the surface has obvious circular bulges (Figure 2B). Moreover, it is parallel with the outer tube of the V unit. The overall shape is similar to the doornail, so we named it as "doornail structure". We speculate that the main role of this doornail structure in *P. dentata* is strengthening the overall structure of coccolith and making it less prone to breakage. Additionally, the circular convex structures of coccolith shells might possess three major uses such as: 1. making the doornail structure sturdier; 2. refracting the light which allows light to enter the cell better; 3. regulating the speed of water entering and leaving the cell.

Previous reports have revealed that coccoliths are a small structure with higher risk to get damaged, especially during the sample preparation process 49, 54, 65, 66. Thus, that's making it highly difficult to study the complete structure of coccolithophore. Till date, there are very few high-resolution images of complete coccolithophores and their supporting materials. We are very fortunate in this regard to capture a relatively complete coccolith of *P. dentata* for the first time. We have carefully observed and explained about the intricate structural and morphological aspects of relatively complete coccolith shell structure of *P. dentata* (Figure 5). Using Figure 5, we have proposed that each of the coccolith's double discs crosses with each other and they are fixed to each other like a bolt. Presence of a latch structure on the outer shell of *P. dentata* might propose its role in the cell division. The double discs of each coccolith will be gradually separated as the protoplasts expand, just like opening the bolts. These expanding bolts protect the coccolith from damaged during the splitting process. We strongly believe the structural and morphological aspects of novel 3D structures of *P. dentata* coccolith can be used further understanding about the coccolithophores.

Along with acidification ocean waters are also subjected to eutrophication another anthropogenic problem caused due to over-enrichment of minerals and nutrients, especially effecting the coastal waters. These nutrients mainly include nitrate and phosphates, increased concentrations of these nutrients stimulate primary producers of the aquatic system, especially algae and phytoplankton, which leads to massive algal and phytoplanktonic blooms 67. Thus, we continued our research to study the effects of different concentrations of potassium nitrate (0.25, 0.5, 0.75, and 1 mmol L^-1^, without potassium nitrate as control) on CaCO_3_ accumulation, total lipid, carbohydrate, and protein contents in *P. dentate*. When KNO_3_ concentration was 0.75 mmol L^-1^, *P. dentata* accumulated the highest CaCO_3_ content, with about 10.01 % of dry weight on day 10 (Figure 2 A/B). However, the control group (0 mmol L^-1^) had the lowest calcium content with about 3.97% (Figure 2B). Under the effect of different concentrations of potassium nitrate, the CaCO_3_ accumulation rate in *P. dentata* was different (Figure 2B). To verify the results, we have also observed the cell surface calcification of the day 10 samples using SEM (Figure 3). The calcification trends of *P. dentata* shown in the SEM were almost identical to the calcium content trends of *P. dentata* (Figure 2B). Previous studies have reported that the calcium content of *Pleurochrysis carterae* is 10% 57, 68. *P. carterae* exhibited two phases, calcifying phase and non-calcifying phase 31, 49. *P. carterae* do not accumulate coccolith on the cell surface in its early stages of growth or when it is undernourished. *P. carterae* was surrounded by calcium carbonate shells when it was well-nourished or in its later stages of growth stage 31.

Microscopic observations of day 10 *P. dentata* control (no potassium nitrate) cultures exhibited lesser quantities of calcium carbonate accumulation on its cell surface (Figure 3 A-1). Results obtained in our study has shown that *P. dentata* cultured with 0 to 0.75 mmol L^-1^ concentration of KNO_3_ showed an increasing rate of CaCO_3_ accumulation. In accordance with the literature, we also believe that KNO_3_ could enhance the accumulation of CaCO_3_ on the algal cell surface, especially when *P. dentata* cultured with 0.75 mmol L^-1^ exhibited highest calcium accumulation on its cell surface. These results suggest that 0.75 mmol L^-1^ of KNO_3_ supported *P. dentata* during synthesizing of calcium carbonate shell as K^+^ is an activator for a variety of enzyme which plays an important role during the cellular metabolism.

Interestingly, *P. dentata* cultured with 1 mmol L^-1^ concentration of KNO_3_, were found to grow independently (Figure 3 E-1); Whereas, *P. dentata* cultured with KNO_3_ concentration less than 1 mmol L^-1^ exhibited agglomeration. Similarly, *P. dentata* cells entered into its active stage in advance when cultured with 1 mmol L^-1^ concentration of KNO_3_. At the same time, affecting the CaCO_3_ accumulation and cell size. This confirmed that higher concentrations of K^+^ potentially could disrupt the calcium uptake 69. Results obtained in our study concludes that adding extra 0.75 mmol L^-1^ potassium nitrate to the f/2 medium allows *P. dentata* to accumulate more calcium carbonate on its surface. During 10 days of cultivation, the total protein content of *P. dentata* increased with increasing concentrations of potassium nitrate (Figure 6A). Otherwise, the lipid and carbohydrate contents of *P. dentata* decreased with increasing concentrations of KNO_3_ (Figure 4A, 5A). To verify these results, we have performed the FTIR technique to detect the total content of protein, lipid and carbohydrates. A comparison between both the results (Table 1 and Figure 6B) has conveyed a positive correlation between the content of protein and the KNO_3_ content, followed by a negative correlation between KNO_3_ content and lipid, carbohydrate contents, respectively. It is well known that K^+^ is an essential micronutrient and it plays a crucial role as an activator for a variety of enzyme involved in controlling the fate of cellular metabolism. Especially, K^+^ plays a crucial role in photosynthesis, nitrogen metabolism and improve nitrogen absorption and utilization, regulation of cellular osmotic pressure and plant growth regulation 70. Earlier studies reported that plants synthesize more proteins when nitrogen is abundant, thus, further promoting cell division and growth. When the potassium nitrate concentration is 1 mmol L^-1^, *P. dentata* is more fragmented (Figure 3 E-1).

## Conclusion

In our present study, we have tried to understand the effect of KNO_3_ on *P. dentata* especially on cellular accumulation of CaCO_3_, and biological macromolecules such as lipid, protein and carbohydrates respectively. Results obtained from this study have shown that *P. dentata* cultured with higher concentrations of KNO_3_ accumulated higher rates of protein and lower rates of lipids and carbohydrates respectively. Our study also reports that *P. dentata* cultured with higher concentrations of KNO_3_ inhibits the cell size of *P. dentata* and CaCO_3_ accumulation in shells. Contrastingly, higher concentrations of KNO_3_ had a positive effect on its cell growth. Later, we have extended our current study to understand the structural and morphological characteristics of *P. dentata* coccolithophores. To the extent of our knowledge this is the first report showing the complete coccolithophore structure (devoid of protoplast) of *P. dentata*. Based on the observations made from the SEM analysis we have proposed the structural arrangement of coccolithophore shells. Using these observations we have tentatively proposed the “doornail” and the “double-disc” structures of the developing coccolithophores of *P. dentata*. We strongly believe that results obtained in our current study enhances the present day's knowledge on *P. dentata* coccolithophores and effect of micronutrients. These findings can be further implemented to understand the favorable culture conditions for the growth and development of *P. dentata*. Our future studies are focussed on understanding the effect of other micronutrients on growth and development of *P. dentata.* and cellular accumulation of valuable bio-products.

## Supplementary Material

Supplementary figures and tables.

## Figures and Tables

**Table 1:** Transmittance rate of three products under different concentrations of KNO_3_

	KNO_3_ (mmol L^-1^)				
Absorption band	0	0.25	0.5	0.75	1
Lipid (2953 cm^-1^)	95.01 %	96.31 %	96.44 %	98.75 %	99.81 %
Protein (1649 cm^-1^)	97.24 %	97.21 %	97.20 %	96.34 %	95.98 %
Carbohydrate (1072 cm^-1^)	94.64 %	94.99 %	96.28 %	96.40 %	96.42 %
