# Supplementary material for: Effect of KNO3 on Lipid Synthesis and CaCO3 Accumulation in Pleurochrysis dentata Coccoliths with a Special Focus on Morphological Characters of Coccolithophores
Source: Int J Biol Sci. 2019 Nov 1;15(13):2844–58. doi: 10.7150/ijbs.35664 (PMC6909966; doi:10.7150/ijbs.35664)
Supplement: Supplementary file 1 — Supplementary figures and tables. [file ijbsv15p2844s1.pdf]

Effect of KNO_3 on Lipid Synthesis and $CaCO_3$ Accumulation in *Pleurochrysis dentata* Coccoliths With a Special Focus on Morphological Characters of Coccolithophores

Xuantong Chen[1, 2], Ayyappa Kumar Sista Kameshwar[1], Chonlong Chio[1], Fan Lu[3], Wensheng Qin[1]*

[1]Department of Biology, Lakehead University, 955 Oliver Road, Thunder Bay, Ontario, P7B 5E1, Canada.
[2]Faculty of Natural Resources Management, Lakehead University, 955 Oliver Road, Thunder Bay, Ontario, P7B 5E1, Canada. [3]School of Civil Engineering, Architecture and Environment, Hubei University of Technology, Wuhan, China, 430068. *Email address: wqin@lakeheadu.ca

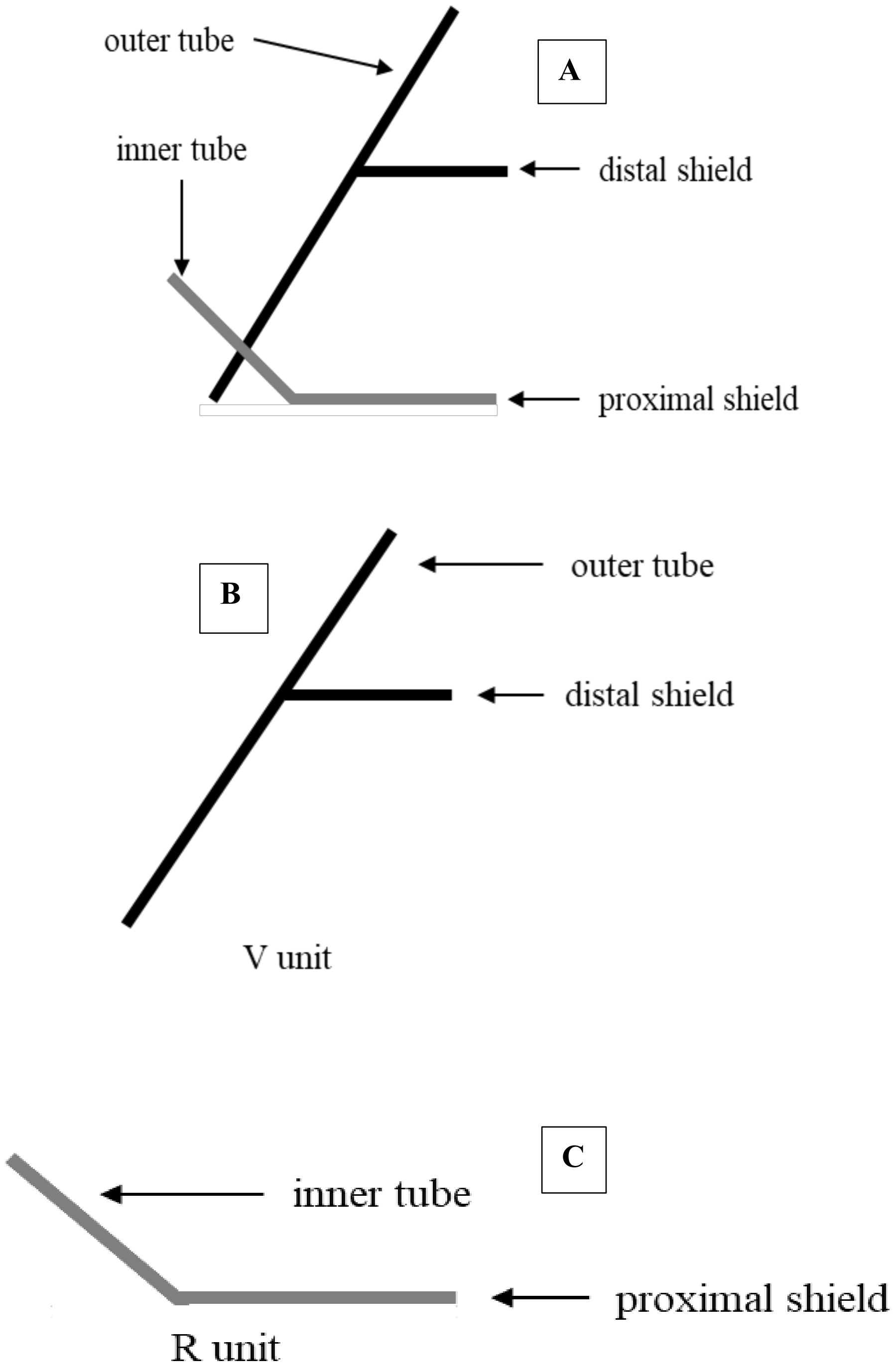

Fig. S1: Coccolith structure reported in previous research studies: **A:** V unit Side view. **B:** R unit Side view. **C:** V unit and R unit structure side view.

<u>Sequence-S1</u>

The sequencing results of *Pleurochrysis* sp. 18s rRNA (**DNA**) sequence:

>Pleurochrysis sp. *18srRNA* Sequence

>P. dentata LU118SrRNA

NNNNNNNNNNNNNNNNNNGTACCTTACNACTTGGATACCCGTAGTAATTCTAGAGC
TAATACATGCAGGAGTTCGTGCGCGGCTCGTCCGCGCGATGTATTTATTAGATAA
GAGACCAACCCGCCTCGTGCGGTTGCGTGCCGAGTCATAATAACTGTTCGAATCGCA
TGGCTCTGACGCCGGCGATGGTTCATTCAAATTTCTGCCCTATCAGCTTTCGATGGTA
GGATAGAGGCCTACCATGGCGTTAACGGGTAACGGGGAATTAGGGTTCGATTCCGG
AGAGGGAGCCTGAGAGATTGNNGCTANNGNNNCCACAGTCCAGAGNNNNGAATGG
CAGTTTNCAGGCGCGTAAATNNNNNNNNNNTGCCCNNNNNNNNNNNNGNNNTAANTNC
CTGACACAGGGAGGTAGTGACAANNGAANATANACAATACAGGGCCATCTTGGTCT
TGTAATTGGAATGAGTACAATTTACATCTCTTCACGAGGATCAATTGGAGGGCAAGT
CTGGTGCCAGCAGCCGCGGTAATTCCAGCTCCAATAGCGTATATTAAAGTTGTTGCA
GTTAAAACGCTCGTAGTCGGATTTCGGGTCGGTTGCGCCGGTCTGCCGATGGGTATG
CACTGGTGGAGTCGGCCTTTCTTCCGGAGACCGCGCCTCCTCTTAGCTGAGCGGGTG
CGGGAGACGGATCGTTTACTTTGAAAAAATCAGAGTGTTTCAAGCAGGCAGCTCGCT
CTTGCATGGATTAGCATGGGATAATGAAATAGGACTCTGGTGCTATTTTGTTGGTTT
CGAACACCGGAGTAATGGTCAACAGGGACAGTCAGGGGCACTCGTATTCCGCCGAG
AGAGGTGAAATTCTCAGACCAGCGGAAGACGAACCACTGCGAAAGCATTTGCCAGG
GATGTTTTCACTGATCAAGAACGAAAGTTAGGGGATCGAAGACGATCAGATACCGT
CGTAGTCTTAACCATAAACCATGCCGACTAGGGATTGGAGGCTGTTCCTTTTGTGAC
TCCTTCAGCACCTTTCGGGAAACTAAAGTCTTTGGGTTCCGGGGGGGAGTATGGTCG
CAAGGGCTGAAACTTAAAGGAATTGACGGAAGGGGCACCACCNAGGGAGTGGAGC
CTGGCGGCTTAATTTGACTCAACACGGGGGAAACTTANCCAGGTNCCAGACATTGTG
ANGGATTGACAGANTTGAGNNANGCTCTTTCTTGATTNCGATGGGNTGGTGGTGCAT
GGCCNNGTTCTAAAGTTGGTGGAGTGANNTTTGTCNNTGGTTAATTCCGTTNNNNNA
ANCGAANCNNNNNNNNNNNNNNNNNNNNNNNNNNNNNGANNGACCGCANNGCCCCC
CAAACACCTTCGCGAACACTCNCGTTGGCGTTGAGCTTCTTAGAGGGACAACTTGTC
TTCAACAAGTGGAAGTTTGCGGCAATAACAGGTCTGTGATGCCCTTAGATGTTCTGG
GCCGCACGCGCGCTACACTGATGCACTCAACGAGTTTTCTCCTTGGCCGAGAGGTCC
GGGTAATCTTTTGAAATTGCATCGTGATGGGGATAGATTATTGCAATTATTAATCTTC
AACGAGGAATTCCTAGTAAGCGCATGTCATCAGCGTGCGTTGATTACGTCCCTGCCC
TTTGTACACACCGCCCGTCGCTCCTACCGATTGAATGATCCGGTGAGGCCCCCGGAC
TGTGGCAACGCAGTTGGTTCTCCAGCCGCGATGCCGCGGGAAGCTGTCCAAACCTTA
TCATTNGAGANNNNNNNNNNNNNNNNNN

Table-S1: Lists the NCBI BLAST results for the *Pleurochrysis sp.* 18srRNA gene sequences:

Description	Max score	Total score	Query cover	E value	Perc. ident	Accession
Pleurochrysis dentata HAP6	1483	2729	88%	0	96.89%	AJ544121.1
Chrysotila sp. 1 JIK-2013 strain A13 801	1478	2727	88%	0	96.78%	KJ020926.1
Chrysotila sp. 1 JIK-2013 strain A13 799	1478	2727	88%	0	96.78%	KJ020925.1
Chrysotila sp. 1 JIK-2013 strain A13 798	1478	2727	88%	0	96.78%	KJ020924.1
Chrysotila sp. 1 JIK-2013 strain A13 797	1478	2727	88%	0	96.78%	KJ020923.1
Chrysotila sp. 1 JIK-2013 strain A13-255	1478	2727	88%	0	96.78%	KF696662.1
Pleurochrysis dentata strain CCAP 944/2	1478	2721	88%	0	96.78%	KJ756811.1
Pleurochrysis placolithoides ALGO HAP59bis	1478	2703	88%	0	96.78%	AM490977.2
Pleurochrysis sp. MBIC10549	1478	2727	88%	0	96.78%	AB183616.1
Pleurochrysis dentata CCAP 904/1	1474	2620	85%	0	96.67%	MG022746.1
Pleurochrysis sp. ALGO	1472	2703	88%	0	96.67%	AM490975.2
Pleurochrysis sp. NMBjih026	1472	2717	88%	0	96.67%	EF208116.1
Pleurochrysis sp. MBIC10443	1472	2655	88%	0	96.67%	AB183596.1
Chrysotila carterae CCAP 961/1	1461	2669	90%	0	96.45%	MG022757.1
Chrysotila stipitata A13-253	1461	2675	90%	0	96.45%	KF728655.1
Chrysotila stipitata A13-250	1461	2675	90%	0	96.45%	KF728654.1
Chrysotila stipitata A13-110	1461	2675	90%	0	96.45%	KF696663.1
Pleurochrysis scherffelii ALGO HAP11	1461	2671	89%	0	96.45%	AM490978.2
Pleurochrysis carterae HAP1	1461	2666	89%	0	96.45%	AJ544120.1
Chrysotila pseudoroscoffensis CCAP 912/1	1456	2612	88%	0	96.34%	MG022748.1
Pleurochrysis carterae KMMCC H-19	1456	2653	90%	0	96.34%	HQ877918.1
Pleurochrysis pseudoroscoffensis ALGO HAP48	1456	2608	88%	0	96.34%	AM490973.2
Pleurochrysis gayraliae partial ALGO HAP10	1456	2603	88%	0	96.34%	AM490972.2

Table-S2: Statistical significance analysis of KNO_3 concentration and its effect on $CaCO_3$ accumulation in *Pleurochrysis dentata*

Concentration mmol·L^{-1}	N	alpha = 0.05				
		1	2	3	4	5
0.00	3	.039700				
0.25	3		.061800			
0.50	3			.070400		
1.00	3				.078000	
0.75	3					.100100
Significance		1.000	1.000	1.000	1.000	1.000

Table-S3: Statistical significance analysis of KNO_3 concentration and its effect on total lipid content in *Pleurochrysis dentata.*

Concentration mmol·L^{-1}	N	alpha = 0.05				
		1	2	3	4	5
1.00	3	.13670000				
0.75	3		.19000000			
0.50	3			.25670000		
0.25	3				.31980000	
0.00	3					.33610000
Significance		1.000	1.000	1.000	1.000	1.000

Table-S4: Statistical significance analysis of KNO_3 concentration and its effect on total carbohydrate content in *Pleurochrysis dentata.*

Concentration mmol·L^{-1}	N	alpha = 0.05				
		1	2	3	4	5
1.00	3	.21077110				
0.75	3		.22264591			
0.50	3			.27014383		
0.25	3				.30139005	
0.00	3					.33701315
Significance		1.000	1.000	1.000	1.000	1.000

Table-S5: Statistical significance analysis of KNO_3 concentration and its effect on total protein content in *Pleurochrysis dentata.*

Concentration mmol·L^{-1}	N	alpha = 0.05				
		1	2	3	4	5
0.00	3	.27212107				
0.25	3		.28424314			
0.50	3			.29030301		
0.75	3				.31143105	
1.00	3					.32868696
Significance		1.000	1.000	1.000	1.000	1.000

Preparation of 18S rRNA cDNA for polymerization and sequencing:

a) Firstly, we have used EZ-10 Spin column plant RNA mini-preps kit BioBasic® Canada Inc for isolating the total mRNA. Later we have used 5X All-in-one RT Master Mix to convert the above isolated mRNA to cDNA.
b) We have polymerized the 18S gene products using the standard 18S rRNA forward: GAAACTGCGAATGGCTCATT and reverse: CCTTCTGCAGGTTCACCTAC primers and above isolated *P. dentata* cDNA sequences using Bio-Rad® PCR machine using the standard conditions.
c) The polymerized 18S rRNA gene products obtained were characterized using 1% agarose gel electrophoresis to characterize the obtained PCR product. Finally, the above characterized 18S rRNA product was retrieved using the Gel/PCR DNA fragment extraction kit.
d) The gene product obtained from the gel extraction kit is further sent to the sequencing company.

Sequence-S2

The sequencing results of *Pleurochrysis* sp. cDNA sequence coding for 18s rRNA

>Pleurochrysis sp. 18srRNA (**cDNA**) Sequence

TTGGTGTGTTTATTGATGGTACTTACTACTTGGATACCCGTAGTAATTCTAGAGCTAATAC
ATGCAGGAGTTCGTGCGCGGCTCGTCCGCGCGATGTATTTATTAAATAAGAGACCAAC
CCGCCTCGTGCGGTTGCGTGCCGAGTCATAATAACTGTTCGAATCGCATGGCTCTGACGC
CGGCGATGGTTCATTCAAATTTCTGCCCTATCAGCTTTCGATGGTAGGATAGAGGCCTAC
CATGGCGTTAACGGGTAACGGGGAATTAGGGTTCGATTCCGGAGAGGGAGCCTGAGAGA
TGGCTACCACATCCAAGGAAGGCAGCAGGCGCGTAAATTGCCCGAATCCTGACACAGGG
AGGTAGTGACAAGAAATAACAATACAGGGCCATCTTGGTCTTGTAATTGGAATGAGTAC
AATTTACATCTCTTCACGAGGATCAATTGGAGGGCAAGTCTGGTGCCAGCAGCCGCGGA
AATTCCAGCTCCAATAGCGTATATTAAAGTTGTTGCAGTTAAAACGCTCGTAGTCAGATT
TCAGGTCGGTTGCTCCGGTCTGCCGATGGGTATGCACTGGTGGAGTCAGCCTTTCTTCCG
GAGACCGCGCCTCCTCTTAGCTGAGCGGGTGCGGGAGACGGATCGTTTACTTTGAAAAA
ATCAGAGTGTTTCAAGCAGGCAGCTCGCTCTTGCATGGATTAGCATGGGATAATGAAATA
GGACTCTGGTGCTATTTTGTTGGTTTCGAACACCGGAGTAATGGTCAACAGGGACAGTCA
GGGGCACTCGTATTCCGCCGAGAGAGGTGAAATTCTCAGACCAGCGGAAGACGAACCAC
TGCGAAAGCATTTGCCAGGGATGTTTTCACTGATCAAGAACGAAAGTTAGGGGATCGAA
TACGATCAATCCCGTCGTAGTCTTACCCATTAAACCATGCCCGACTAGGGAATTGTTCTC
TTCTGACTTTCTCTTCTCTACTGATAGGTTTGGACAGCTTCCCGCGGCATCGCGGCTGGAG
AACCAACTGCGTTGCCACAGTCCGGGGGCCTCACCGGATCATTCAATCGGTAGGAGCGA
CGGGCGGTGTGTACAAAGGGCAGGGACGTAATCAACGCACGCTGATGACATGCGCTTAC
TAGGAATTCCTCGTTGAAGATTAATAATTGCAATAATCTATCCCCATCACGATGCAATTT
CAAAAGATTACCCGGACCTCTCGGCCAAGGAGAAAACTCGTTGAGTGCATCGTTGTAAC

GCGCGTGCGGCCCAAAACATCTAAGGGCATCACAGACCTGTTATTGCCGCAAACTTCCAT
TGTTGAAGACAAGTTGTCCCTCTAAAAAGCTCAACGCCAACGGAATGTTCGATAAACTAT
TTTTCAGGATGGGGGCTCTTTCGTTAACGGAATTAACCAAACCAATCACTCCACCAACTA
AGAACGGCCATGCACAACCACCCATCGTATCAAGAAAGAGCTCTGAATCTGTCAATCCT
CACAATGTCTGGACCAGGCTGGTTATGGCGTGTTGAGTCAAATTAAGCCGCAGGCTCCAC
TCCTGGTGGTGCCCTTCCGTCAATTCCTTTTAAGTTTCAGCCTTGCGACCATACTCCCCCC
GGAACCCAAAGACTTTTAGTTTCCCGAAAGGTGCTGAAGGAGTCACAAAAGGAACAGCC
TCCAATCCCTAGTCGGCATGGTTTATGGTTAAGACTACGACGGTATCTGATCGTCTTCGA
TCCCCTAACTTTTCGTTCTTGATCAGTGAAAACATCCCTTGGCAAATGCTTTCGCAGTGGT
TCGATCTTCCGCTGGGTCTGAGAATTTTCACCTTCTCTCGGGCGGAATTACGAGTGCCCCT
GACTGTTCCCTGTTGGACCATTACTTCCGGTGTTCGAAACCACAAATATCACCAGAAGTT
CCTATTTCATAATCCATGCTATATCATGCAAAGCCGGTGCTGGCCTGCTGTGAACACTTCT
GAATTTTTTTTCCAAGTTAACCGAATCGGATCTCTCCTTACCGCCCCGTCTCCATGCTATT
AAAATGT

Table-S6: Lists the NCBI BLAST results for the *Pleurochrysis sp.* 18srRNA-cDNA (mRNA coding for 18srRNA converted to cDNA) sequences:

Description	Max score	Total score	Query cover	E value	Perc. ident	Accession
**Pleurochrysis dentata isolate HAP6**	1657	3174	95%	0	98.41%	AJ544121.1
Pleurochrysis dentata isolate CCAP 904/1	1652	3071	92%	0	98.31%	MG022746.1
Chrysotila sp. 1 JIK-2013 strain A13 801	1652	3174	96%	0	98.31%	KJ020926.1
Chrysotila sp. 1 JIK-2013 strain A13 799	1652	3174	96%	0	98.31%	KJ020925.1
Chrysotila sp. 1 JIK-2013 strain A13 798	1652	3174	96%	0	98.31%	KJ020924.1
Chrysotila sp. 1 JIK-2013 strain A13 797	1652	3174	96%	0	98.31%	KJ020923.1
Chrysotila sp. 1 JIK-2013 strain A13-255	1652	3171	95%	0	98.31%	KF696662.1
Pleurochrysis dentata strain CCAP 944/2	1652	3169	96%	0	98.31%	KJ756811.1
Pleurochrysis sp. MBIC10549	1652	3174	96%	0	98.31%	AB183616.1
Pleurochrysis sp. ALGO	1646	3147	95%	0	98.20%	AM490975.2
Pleurochrysis sp. NMBjih026	1646	3163	95%	0	98.20%	EF208116.1
Pleurochrysis placolithoides ALGO HAP59bis	1640	3147	95%	0	98.10%	AM490977.2
Pleurochrysis sp. MBIC10443	1629	3097	95%	0	97.88%	AB183596.1
Chrysotila carterae isolate CCAP 961/1	1624	3104	95%	0	97.78%	MG022757.1
Chrysotila stipitata strain A13-253	1624	3113	96%	0	97.78%	KF728655.1
Chrysotila stipitata strain A13-250	1624	3113	96%	0	97.78%	KF728654.1
Chrysotila stipitata strain A13-110	1624	3113	96%	0	97.78%	KF696663.1
Pleurochrysis scherffelii ALGO HAP11	1624	3108	95%	0	97.78%	AM490978.2
Pleurochrysis sp. CCMP 875	1622	3095	96%	0	97.98%	AJ246265.1

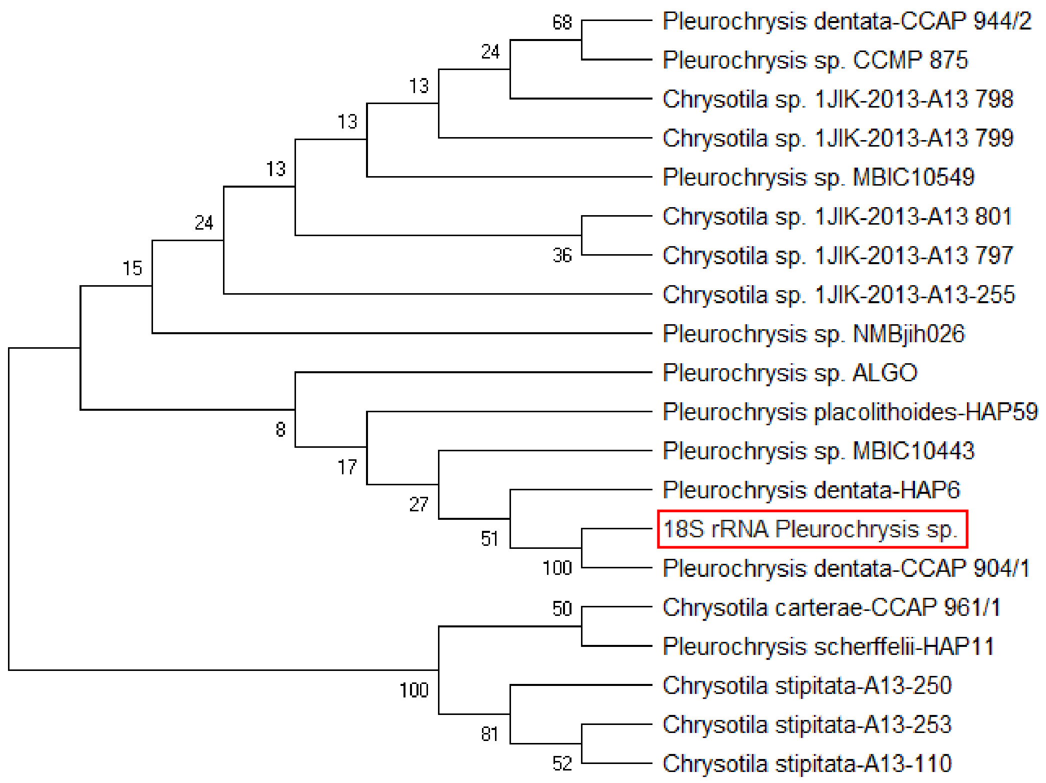

Fig S2: The phylogenetic analysis of the BLAST results obtained for the *Pleurochrysis sp.* 18S rRNA (cDNA) sequence.
